# Nutritionally adequate, healthy, and climate-friendly diets following the Nordic Nutrition Recommendations 2023: an optimization study for Norway

**DOI:** 10.3389/fnut.2025.1485287

**Published:** 2025-05-14

**Authors:** Julie Lengle, Chi Zhang, Arnoldo Frigessi, Lene Frost Andersen

**Affiliations:** ^1^Department of Nutrition, University of Oslo, Oslo, Norway; ^2^Department of Biostatistics, University of Oslo, Oslo, Norway

**Keywords:** sustainable diets, diet optimization, greenhouse gas emissions, dietary guidelines, Nordic Nutrition Recommendation, environmental impact reduction, nutritional adequacy, Nordic diets

## Abstract

**Background:**

Reducing red meat consumption is an effective tactic for decreasing environmental impact of diets while maintaining nutritional adequacy, healthiness, and overall consumer acceptability. Still, dietary change in favor of plant foods is a controversial climate mitigation measure, especially in the Nordic region where agri-food heritage is linked to ruminant husbandry.

**Objective:**

In this study we aimed to explore sustainable diets for the Norwegian context by (1) investigating the environmental impacts of nutritionally optimized diets following the Nordic Nutrition Recommendations 2023 (NNR2023), (2) estimating potential for environmental impact reduction across scenarios of meat and legume consumption, and (3) identifying nutritional challenges.

**Methods:**

Quadratic optimization was employed to minimize departure from the average observed Norwegian diet while meeting nutrient, health, and carbon footprint constraints. The diet of Norwegian adults was estimated based on results from the national dietary survey Norkost 3. Global warming potential (GWP), freshwater and marine eutrophication, terrestrial acidification, water use, and transformation and use of land were calculated using data from the Norwegian Life Cycle Assessment Food Database version 01. Diets were optimized to meet NNR2023 nutrition and health recommendations for nutrients and food groups. Optimizations were first run without constraints on GWP, for three diet scenarios: (1) nutrients and health-based targets for food amounts (*NNR2023*), (2) nutrients and health-based targets for food amounts with ruminant meat ≥ observed intake (62 g/day) (*Ruminant*), and (3) nutrients and health-based targets for food amounts with legumes content ≥40 g/day (Legumes). Then, GWP constraints were applied in 5% increments until no solution was found. The optimal diet for each scenario was defined as the diet with the largest feasible reduction in GWP (NNR2023+/Ruminant+/Legumes+).

**Results:**

Optimizing the diet to meet nutrient and health constraints alone resulted in a modest decrease in GWP (NNR2023); retaining ruminant meat consumption (Ruminant) impeded the reduction (−9% vs. 0%). Diets following NNR2023 nutrient and health constraints alone were feasible up until a 30% reduction in GWP (NNR2023+). A 35% reduction in GWP was achieved when legumes were added to the diet (Legumes+), while diets retaining 62 g of ruminant meat were not identified beyond a 15% reduction in GWP (Ruminant+). Sodium and selenium were the strongest limiting constraints in all scenarios. Diets with a 40% reduction in GWP were identified when nutrient constraints were lowered from the Recommended Intake to the Average Requirement (NNR2023+/Legumes+). Reductions in GWP coincided with reductions in all measured environmental indicators except marine eutrophication.

**Conclusion:**

The NNR2023 guidelines outline diets that have generally lower environmental impacts than the average Norwegian diet, though outcomes depend on distribution of meat and legume consumption in the diet. Regardless of degree of environmental impact reduction, diets following NNR2023 guidelines will require significant dietary changes compared to observed intake, including an increase in consumption of fruits, vegetables, and grains, and a strong decrease in consumption of red meat, total meat, and discretionary foods. Preventing the model from removing any ruminant meat from the diet limited GWP reduction to 15% and induced considerable changes in intake of other food groups, especially a decrease in other types of meat.

## 1 Introduction

Despite considerable evidence of large potential environmental benefits from a shift toward less meat-intensive diets, global meat consumption continues to rise (1–3). In Norway, per capita meat consumption reached a record high of 79 kg per capita in 2021; over two thirds of this consumption was red meat, whereof approximately 35% was meat from pigs, 25% meat from cattle, and 6% meat from lamb and goats (4). In 2020 the Norwegian Environment Agency released the report Climate Cure 2030 – Measures and Instruments Toward 2030 (5). In this report, the agency highlights a gradual reduction in red meat, supported by an increase in plant-based foods and fish, as the single most promising tactic to reduce emissions from the Norwegian agricultural sector. Further, the Nordic Nutrition Recommendations 2023 (NNR2023) and the Norwegian dietary guidelines (2024) recommend a red meat intake below 350 grams/week (cooked weight), citing evidence of a largely linear relationship between red meat and risk of colon cancer, cardiovascular disease, and type 2 diabetes (6, 7). In this instance, the interests of both environmental and health organizations seem to align.

However, while various national and international authoritative bodies encourage reducing meat production and consumption, several studies indicate high levels of conflict in the interpretation of the associated environmental and health impacts, and what policy initiatives should be considered (8–10). These issues are particularly relevant in Norway, where 74% of employment in agriculture is linked to meat and dairy production (11). Perhaps unsurprisingly, a recent analysis of public hearing responses to the Climate Cure 2030 report identified a strong resistance from most actors to discuss any form of lowering meat production (10). Important actors from the meat and dairy industry have instead issued statements calling for sustainable dietary guidelines that are tailored to the Norwegian context, rejecting the relevance of Nordic and international sustainability guidelines for the Norwegian situation (11). In their arguments, these parties touch on themes such as food security, agriculture throughout the country, value creation, and cultural biodiversity. Though pig and poultry husbandry are large industries in Norway, arguments to preserve meat production often center around the tradition of ruminant husbandry, focusing on the value of grazing livestock for biodiversity and the utilization and maintenance of cultural landscapes (10).

Reducing meat consumption and production is not only a controversial subject among producers. According to the world’s largest survey of public opinion on climate change, dietary change in favor of plant foods is by far the least popular climate mitigation measure (12). Studies show that even dedicated ‘meat reducers’ often struggle to cut back on consumption (13, 14). Some hypothesize that intra-category substitutions, such as replacement of ruminant meat with pork or poultry, will be more acceptable for traditional high meat consumers, due to cultural traits that hinder large reduction in meat consumption (15, 16). However, in Norway, where food heritage is linked not only to meat in general, but to ruminant meat, this may still prove unacceptable (14). To achieve the goals outlined in the Climate Cure 2030 report, additional research is needed to develop strategies for dietary transitions that will be more successful among the target population.

One method for outlining sustainable diets that may be more acceptable for consumers is optimization-based modeling (17). Still, there are few practical tools available to compare the acceptability of alternative diets and few studies have attempted to maximize acceptability of optimized diets by imposing constraints based on specific ‘culturally valued’ food groups. In this study, we aimed to apply quadratic optimization to develop environmentally sustainable diets for the adult Norwegian population that follow the NNR2023 guidelines and consider national food culture. We modeled dietary scenarios with incremental reductions in global warming potential under nutrient, health, co-production, and realism constraints, and measured environmental outcomes in terms of six environmental impact categories. Further, we assessed differences in potential for reduction of dietary environmental impacts in diets with the observed level of ruminant meat (62 g/day) compared to diets with lower amounts of ruminant meat and/or increased amounts of legumes.

## 2 Methods

### 2.1 Dietary intake data

Dietary information data was derived from Norkost 3, a Norwegian national dietary survey among adults conducted in 2010–2011 (18). Dietary information was collected from a nationally representative sample of men (*n* = 862) and women (*n* = 925) aged 18 to 70 years (mean age 46 years). Participants completed two randomly distributed 24-h dietary recalls. Interviewers coded and entered all foods and beverages consumed directly into the in-house food and nutrient composition database and calculation system NutriFoodCalc (Department of Nutrition, University of Oslo). Daily means over two consumption days were calculated for each participant. The survey is described in more detail elsewhere (18).

In the present study, mean daily food intake was calculated, representing the average observed diet of a Norwegian adult. Daily energy intake was standardized to 10 MJ to enable comparability of the observed diet in Norkost 3 with the optimized diet. Mean intakes of individual foods were thus proportionally adjusted to meet an energy intake of 10 MJ, corresponding to the approximate daily reference energy requirement of an average adult across sex and age at a moderate physical activity level (6). Nutritional supplements (i.e., vitamin and mineral supplements, fish oils) were excluded from the analysis.

A total of 1,507 foods were included in the average observed diet, including products in both raw/uncooked (e.g., carrots and eggs) and cooked/processed form (e.g., bread and cold cuts). Food items were aggregated into 53 food sub-groups based on nutritional, culinary, and environmental characteristics. Food sub-groups were further aggregated into main groups for the reporting of results. Food sub-groups and main groups are described in Supplementary Table S1.

Data on the nutritional content of foods was sourced from the nutrition calculation system KBS. The nutrient content in each food sub-group was weighted based on the average consumption of its associated single food items in the population, as described previously by Gazan et al. (19).


$$A_{\mathrm{ij}}=\sum_{k=1}^{n_{j}}\frac{x_{\mathrm{kj}}}{\sum_{k=1}^{n_{j}}x_{\mathrm{kj}}}\times a_{\mathrm{ik}}$$

where A_ij_ the content of nutrient i per gram of food sub-group j; n_j_ is the number of food items belonging to food sub-group j; x_kj_ is the quantity of food item k of food sub-group j consumed in the population and a_ik_ is the content of nutrient i in food item k.

### 2.2 Environmental impact data

Environmental impact information for the food items was sourced from the Norwegian Life Cycle Assessment (LCA) Food Database version 01 (20). The data are based on published Life Cycle Assessment (LCA) studies and include system boundaries from farm-to-retail, thereby including primary production, processing and packaging, international (if relevant) and domestic distribution/transportation, and energy use for storage in wholesale and retail. If the original sourced LCA data did not include waste these data gaps were not filled. Values are included for six environmental impact categories (ICs): the global warming potential of greenhouse gases on a 100-year timescale (kg CO_2_-eq); acidification of soils (kg SO_2_-eq); eutrophication of freshwater (kg P-eq) and marine waters (kg N-eq); water use, specifically the consumption of *extracted water* (21)(m^3^); and transformation and use of land (m^2^a). The Norwegian LCA food database version 01 is integrated into the food composition and nutrition calculation system KBS at the Department of Nutrition at the University of Oslo. The coverage of impact category values ranges from 66% for foods in the food group ‘fruit, berries, nuts and seeds’ to 99% for ‘dairy products’ (20). Coverage of environmental impact values for food (in g) and energy intake (in kJ) in the average observed diet of Norkost 3 participants is at present 98%.

As with nutritional content, the weighted environmental impact values for each of the sub-groups were calculated based on the single food items they include. However, to account for uncertainties in the environmental data, foods were further aggregated into 38 environmental groupings prior to calculation of environmental impacts (see Supplementary Table S2). The estimated environmental impacts of these 38 environmental groupings were then assigned to the 53 food sub-groups mentioned above. For example, white bread and wholegrain bread form two separate food sub-groups, due to important nutritional differences. However, as the difference in environmental impacts between these two sub-groups of bread is uncertain, they were aggregated into one group for calculation of environmental impact. The two groups are thus distinct in terms of nutritional value but share the same environmental impact values in this analysis. All environmental impacts associated with food sub-groups were expressed as impact category (IC) values per g of edible food item (i.e., excluding peel, bones).

### 2.3 Diet optimization

#### 2.3.1 Optimization model

Quadratic optimization was used to search for nutritionally adequate, healthy diets that departed as little as possible from the observed population average diet and met increasingly stringent environmental constraints. The decision variables were the intake amounts of the 53 food sub-groups consumed by the population. For each model, the objective function to be minimized was the quadratic deviation (D) from the mean observed intake, of each food sub-group, as follows:


$$\min\;D,D=\sum_{i}^{53}{\left[\frac{{\mathrm{Opt}}_{i}-{\mathrm{Obs}}_{i}}{Obs_{i}}\right]}^{2}$$

where Obs_i_ and Opt_i_ denote the daily consumption of food sub-group (i) in the observed and optimized diets, respectively. This method discourages new diets with large changes in diet composition compared to the observed diet, assuming that deviation from the observed diet is a reasonable proxy for diet acceptability. We minimize D over possible consumptions of the 53 food subgroups, taking into account a number of constraints, as described next.

The optimizations were performed using the IBM CPLEX solver run through the Rcplex package version 0.3–5 of the R statistical software version 4.1.3.

#### 2.3.2 Model constraints

An overview of the nutrient constraints applied in the optimization is presented in Table 1. In order to ensure nutritional adequacy of the proposed diets, lower and upper limits for macro- and micronutrients were enforced. These constraints were based on the NNR2023 recommendations (6). Macronutrient recommendations for fat, protein, and carbohydrates were based on targets for dietary planning purposes. Fatty acid quality was ensured by constraining saturated and n-3 fatty acids and measuring the content of *α*-*Linolenic acid* (ALA), mono-unsaturated fatty acids (MUFA), and poly-unsaturated fatty acids (PUFA). Micronutrient limits were based on recommended intake (RI) values when available; if RI was not available, adequate intake (AI) was used. The RI represents the average daily nutrient intake level that is sufficient to meet the nutritional requirements of nearly all (usually 97.5%) individuals in a particular life-stage group in the general population; the AI has larger uncertainty than RI, but is expected to meet or exceed the needs of most individuals in the life-stage group (6). Constraints were not set for Tolerable Upper Intake Level (UL); nutrient amounts in the final optimized diets were later checked to ensure they did not surpass available ULs. For nutrients with differing recommendations for females and males, an average was taken of the two values, assuming equal sex distribution in the population. Exceptions were made for Vitamin D and selenium. Vitamin D adequacy is difficult to achieve through dietary intake alone, and suboptimal vitamin D-status is common among population groups in Norway that are less exposed to sunlight and do not take vitamin D supplements (22). Constraining vitamin D may have led to unrealistic optimized diets; therefore, as in previous studies, vitamin D content in the diets was measured rather than constrained (23, 24). Preliminary analyses indicated a difficulty in fulfilling the selenium recommendation provided by NNR2023 (AI, average males and females: 82.5 μg/d) without imposing large changes in the diet. The provisional average requirement (AR) of 65 μg/d (average males and females) was thus used instead; this is still higher than the RI given in the previous Nordic Nutrition Recommendations from 2012 (NNR2012) (55 μg/d) and twice that of the AR given in NNR2012 (32.5 μg/d) (25). More information follows in section 2.5 Sensitivity analysis.

**Table 1:** Overview of variables applied as nutrient and environmental constraints in the optimization models (C) or measured (but not constrained) in optimized diets (m) and nutritional composition of the average observed daily diet (per 10 MJ).

	Constraint limit^†^	Observed diet*	Optimized diet
Energy, MJ	10	10	C
Protein, E%	10–20 E%	19	C
Carbohydrates, E%	45–60 E%	46	m
Added sugar, E%	<10 E%	8	C
Dietary fiber, g	≥30	**28**	C
Fat, E%	25–40 E%	37	C
Saturated fatty acids, E%	<10 E%	**13**	C
Trans fatty acids, E%	≤1 E%	0.4	m
n-3 fatty acids, E%	≥1 E%	1.2	C
ALA, E%	≥0.5 E%	0.9	m
MUFA, E%	10–20 E%	14	m
PUFA, E%	5–10 E%	6	m
Vitamin A, RE μg	≥750	816	C
Vitamin E, alfa-TE	≥10.5	15	C
Thiamin (Vitamin B1), mg	≥1	2	C
Riboflavin (Vitamin B2), mg	≥1.6	2.3	C
Niacin, NE	≥16	23.2	C
Vitamin B6, mg	≥1.7	1.9	C
Folate, μg	≥330	**287**	C
Vitamin B12, μg	≥4	8	C
Vitamin C, mg	≥102.5	118.1	C
Vitamin D, μg**	≥10	**6.7**	m
Sodium, mg	≤2400***	**3,248**	C
Potassium, mg	≥3,500	4,510	C
Calcium, mg	≥967	1,108	C
Magnesium, mg	≥325	387	C
Phosphorus, mg	≥520	1950	C
Iron, mg	≥10.8	11.3	C
Zinc, mg	≥11.2	12.5	C
Iodine, μg	≥150	220	C
Selenium, μg	≥65****	**61.2**	C
Copper, μg	≥0.9	1.4	C
Alcohol, g*****	0	**9.3**	C
Environmental constraints			
Global warming potential, kg COe-eq	_	5.2	m/C******
Freshwater eutrophication, g P-eq	_	1.2	m
Marine eutrophication, g N-eq	_	4.9	m
Terrestrial acidification, g SO2-eq	_	55	m
Water use, m3	_	0.6	m
Land use, m2a	_	5.8	m

Bold numbers indicate suboptimal amounts in the observed diet. ^†^Micro- and macronutrient limits based on nutrient recommendations from the Nordic Nutrition Recommendations 2023 (NNR2023). Micronutrient limits are recommended intake (RI) or adequate intake (AI) values, unless otherwise specified (6). ^*^Based on mean daily dietary intake data for adults 18–70 years from the Norkost 3 national dietary surveillance survey 2010–2011 (18). ^**^Vitamin D was not constrained. ^***^The sodium limit recommended by NNR2023 is 2,300 mg. As spices (including salt) have been fixed to observed level in this study, the sodium constraint has been slightly relaxed to the level recommended in The Nordic Nutrition Recommendations 2012 (NNR 2012) (25). ^****^The average requirement (AR, average for males and females) for selenium has been used in this study. The adequate intake (AI) of selenium is 82.5 μg. ^*****^NNR2023 recommend abstinence from alcohol consumption. NNR2012 recommend consumption <10 g/d for women and < 20 g/d for men. The observed consumption is thus below this recommendation, but above the recommendation given in NNR2023 (abstain). ^******^The observed diet was first optimized according to nutrient, health, and realism constraints alone. In this model, global warming potential was measured, not constrained. In the follow models, global warming potential was constrained in 5% reduction increments (−5, −10%, −15% …).

The health-based constraints on food amounts included upper or lower boundaries based on NNR2023: whole grains ≥ 90 g per day, fruit and vegetables ≥ 250 g each per day, juice ≤ 100 g per day, nuts ≥ 20 g per day, vegetable oils and margarine ≥ 25 g per day, milk and dairy products 350–500 g milk-equivalents per day, total fish 300–450 g cooked fish per week (of which ≥ 200 g fatty fish), white meat ≤ current intake, and cooked red meat ≤ 350 g per week. Though NNR2023 only stipulates a “moderate intake” of egg, preliminary analyses with no constraint on egg intake showed a large increase in the content of egg, corresponding to up to 14 eggs per week. A constraint was thus set for egg content in the optimized diets, ≤ 1 egg per day. Since recommendations for meat and fish are provided in cooked weight, these were converted to raw weight using weight change factors (1.45 and 1.21, respectively) (26, 27).

Conversion factors of 1:10 and 1:1 were used for cheese and yoghurt, such that 10 g of cheese corresponded to 100 g milk-equivalents. This is based on recent data from Norwegian dairy production and is in line with the range proposed by NNR2023 (10–20 g cheese per 100 g milk) (6, 28). In addition, a co-production factor of 40:1 for milk-equivalents and ruminant products was included in the model to consider food system co-dependencies. The production of milk and beef are closely linked and a certain consumption of beef must follow intake of milk and dairy products in order to avoid waste. In Norway, approximately 95% of dairy cattle are so-called “hybrid cattle” that produce both milk throughout their lives and meat after slaughter, as well as give birth to offspring (29). In 2030, it is predicted that Norwegian dairy production will include 182,313 cattle and that each cow will produce 8,982 kg of energy-corrected milk (ECM) and 285 kg of meat (28, 30). We considered a conversion factor of 93% for ECM and a meat to carcass weight ratio of 73% (31). This corresponds to a ratio of 40 kg of milk per kg of beef, when applying Eq.:


$$\begin{array}{l} 8,982\;\text{kgECM}\;x\;93\%(\text{conversion factor})\\ x=285\;\mathrm{kg}\;\text{beef}\;x\;73\%(\text{carcass yield}). \end{array}$$

The NNR2023 guidelines recommend a daily consumption of milk and dairy products corresponding to 350–500 g milk-equivalents; this assumes a minimum beef consumption of approximately 9–13 g/d. To prevent the optimization algorithms from including unreasonably high amounts of any single food sub-group, or eliminating others, realism constraints were applied to all models. An upper limit was set for food sub-groups corresponding to the 95^th^ percentile of the intake distribution in the observed diet and a lower limit of 0.1 times the average observed intake. Further, water, coffee, tea, and spices were fixed to observed level due to their secondary role in the diet and in order to maintain acceptability of the diet. Due to spices being fixed to observed level, the sodium constraint in the model was increased to 2,400 mg/d (based on NNR2012, compared to 2,300 mg/d as given in NNR2023). The decision to fix spice intake to observed level and relax the sodium constraint was made due to both uncertainty in salt consumption estimates from the Norkost 3 survey and to prioritize acceptability of the diets (e.g., discourage removal of spices from the diet, replacement of bread with raw whole grains). See section 2.5 Sensitivity analysis.

The observed diet was first optimized in terms of nutrition and health, with no environmental constraints. Then, stepwise constraints were applied for global warming potential (GWP) in 5% increments, until results indicated that no solution was feasible, i.e., no diet satisfying all constraints could be found. The optimal diet was defined as the diet meeting all constraints at the lowest GWP. Limiting nutrients were identified, defined as those nutrients whose values were very close to reaching, or exactly at either lower or upper constraint limits. These nutrients still fulfill imposed constraints, but were active constraints in the optimization (i.e., difficult to meet, such that fulfillment of the constraint influenced the optimization outcome).

#### 2.3.3 Model scenarios

Due to the high GWP associated with ruminant meat, large changes in content of this food group are expected when imposing GWP constraints. Further, due to the nature of the objective function, foods that are consumed in very small amounts (e.g., legumes) in the observed diet are unlikely to be modified appreciably by the optimization model, limiting the opportunities of change. To examine the impact of meat distribution on the optimization outcome, optimization models both with and without GWP constraints were run for three scenarios (Table 2): NNR2023, Ruminant, and Legumes. NNR2023 includes all constraints, as detailed above, with no adjustments. Ruminant includes the same constraints as NNR2023 but imposes a lower limit for intake of ruminant meat, such that content of ruminant meat in the optimized diet may not be lower than observed intake (62 g/day, raw weight). Legumes includes the same constraints as NNR2023 but imposes a modest intake of pulses of legumes, along with elements from the Danish Food-Based Dietary Guidelines (FBDG) (2020) (32). Constraints on daily fruit and vegetable intake were increased to ≥300 g each, inclusion of pulses and legumes ≥40 g/day was forced, and an additional constraint on total meat intake ≤ 350 g cooked weight per week was imposed.

**Table 2:** Overview of health-based constraints for food amounts (g/day) in the NNR2023, Ruminant and Legumes scenarios, compared to amounts in the average observed daily diet (g/10 MJ).

	Consumed amount	Constraint limit		
	Observed diet*	NNR2023	Ruminant	Legumes
Whole grains, g	70	≥90	≥90	≥90
Fruit, g	200	≥250	≥250	≥300
Juice, g	120	≤100	≤100	≤100
Vegetables, g	180	≥250	≥250	≥300
Pulses and legumes, g	4	-	-	≥40
Nuts, g	4	≥20	≥20	≥20
Vegetable oils and margarine, g	16	≥25	≥25	≥25
Milk and dairy products, g**	790	350–500	350–500	350–500
Total fish, g	80	52–78	52–78	52–78
Fatty fish, g	28	≥35	≥35	≥35
Eggs, g	27	≤55	≤55	≤55
Total meat, g	165	-	-	≤73
White meat, g	39	≤39	≤39	≤39
Red meat, g	122	≤73	≤73	≤73
Ruminant meat, g	62	-	≥62	-

Food amounts in raw weight. Optimization scenarios: NNR2023, health constraints based on NNR2023 with no adjustments; Ruminant, additional lower limit for intake of ruminant meat (≥ observed intake); Legumes, lower limit for intake of pulses and legumes (≥40 g) and elements from the Danish FBDG (2020) (32) (fruit and vegetables ≥ 300 g each; total meat ≤350 g cooked weight/week). ^*^Based on mean dietary intake data for adults 18–70 years from the Norkost 3 survey 2010–2011 (18). ^**^Milk-equivalents, using a conversion factor of 1:10 (10 grams of cheese per 100 g of milk) (6, 28). Does not include dairy fats (e.g., butter).

Incremental GWP constraints were then imposed separately for each scenario. The models with GWP constraints will henceforth be referred to as NNR2023+, Ruminant+, and Legumes+.

### 2.4 Diet acceptability

As a proxy for acceptability, a diet departure score (Δdiet) was used to quantify similarity between the average observed diet and the optimized diets. Similarity between optimized diets without GWP constraints and at the maximum feasible GWP reduction were also compared, to adjust for the inevitable departure from the observed diet imposed by changes to meet nutritional and health recommendations. The diet departure score was represented by the average relative deviation from the observed diet (across food sub-groups), and was calculated by


$$\text{Δdiet}(\%)=\frac{1}{53}\sum_{j=1}^{53}|\frac{x_{j}-x_{\mathrm{obs},j}}{x_{\mathrm{obs},j}}|\times 100$$

where x_j_ is the amount of food sub-group in the optimized diet and x_obs,j_ is the amount of food sub-group in the average observed diet (or in the optimized diet chosen for comparison).

### 2.5 Sensitivity analysis

A series of independent sensitivity analyses were performed to examine the impact of methodological choices on model outcomes. First, sensitivity analyses were conducted to investigate the impact of nutrient constraints. In the first sensitivity analysis, all nutrient constraints were lowered to the AR to explore further climate impact reduction potential. The AR represents the average daily nutrient intake level that is estimated to meet the requirements of half the individuals in a population (6). In the second sensitivity analysis, the models were run with the selenium constraint increased from the AR (65 μg/d) to the AI (82.5 μg/d). In the third sensitivity analysis, the upper limit for sodium intake was lowered to the updated NNR2023 recommendation of 2,300 mg.

Then, sensitivity analyses were conducted to investigate the impact of food-group constraints and input data on outcomes. In the fourth sensitivity analysis, the upper intake constraint for white meat was removed. The overall science advice provided by NNR2023 is to limit white meat intake to current level or lower, and instead replace meat consumption with increased consumption of plant foods and fish from sustainably managed stocks. However, this advice is based on environmental factors alone. In this sensitivity analysis, we assess the impact of allowing higher quantities of white meat on the optimization outcomes. In the fifth sensitivity analysis, ruminant meat was split into multiple sub-categories including unprocessed/processed beef, lamb, and ruminant game. Scenarios were run both with and without a co-production constraint linking beef and dairy to assess its impact on distribution of ruminant meat. In the final sensitivity analysis, scenarios were run separately for four population sub-groups. The survey population was split into four quartiles based on daily total meat intake (45 g, 118 g, 186 g, 309 g) and average daily consumption of food sub-groups was calculated for each sub-group individually.

## 3 Results

### 3.1 Nutritional content, environmental impact, and food composition of the observed diet

Values for all nutrients and environmental indicators in the observed diet are presented in Table 1 and average food composition is presented in Table 3. The average observed diet was lower than recommended in intake of dietary fiber, folate, vitamin D, and selenium, and contained too much saturated fat and sodium. The GWP of the average observed intake of Norwegian adults was 5.2 kg CO_2_-eq/10 MJ/day.

**Table 3:** Content of main food groups (g/10 MJ) and environmental impacts (…) per 10 MJ in the average observed Norwegian diet and diet scenarios optimized to meet NNR2023 (6) health and nutrient guidelines, and incremental global warming potential constraints (0, −5%, −10, −15%…).

	Observed diet^†^	NNR2023	Ruminant	Legumes	NNR2023+	Ruminant+	Legumes+
Bread	192	185	161	162	204	203	203
Other grains	40	77	73	71	114	167	159
Cakes, cookies	41	71	82	67	73	28	4
Potatoes	76	139	137	124	187	122	226
Vegetables	165	270	269	300	250	250	300
Fruit and berries	200	313	343	300	250	250	300
Milk	278	345	344	342	412	411	414
Other dairy	80	108	102	100	45	47	16
Cheese	43	5	5	10	4	4	5
Nuts and seeds	4	21	21	21	21	24	31
Legumes	4	6	6	40	7	28	72
Eggs	27	55	55	55	55	55	55
Fish	80	78	78	78	78	78	69
Ruminant meat	62	42	62	46	13	62	12
Pork	60	30	10	27	56	11	6
White meat	38	38	38	38	16	4	4
Other meat	5	2	2	2	2	1	1
Fats, plant-based	16	25	25	25	25	25	25
Fats, animal-based	16	2	2	3	2	2	2
Juice	120	100	100	100	12	12	12
Other beverages	397	223	255	257	26	26	26
Sweets, snacks	23	25	27	32	7	2	2
Water, coffee, tea	2,236	2,236	2,236	2,236	2,236	2,236	2,236
Other	32	5	5	5	5	5	4
Environmental measures							
Global warming potential, kg COe-eq	5.2	4.7 (−9%)*	5.1 **(0%)**	4.8 **(−8%)**	3.6 **(−30%)**	4.4 **(−15%)**	3.4 **(−35%)**
Freshwater eutrophication, g P-eq	1.2	1.1 (−4%)	1.2 (+3%)	1.1 (−3%)	0.9 (−22%)	1.0 (−13%)	0.8 (−29%)
Marine eutrophication, g N-eq	4.9	5.3 (+7%)	5.4 (+10%)	5.2 (+6%)	5.0 (+2%)	5.3 (+8%)	4.9 (−1%)
Terrestrial acidification, g SO2-eq	55.0	47.1 (−14%)	53.5 (−3%)	47.8 (−13%)	35.0 (−36%)	48.1 (−13%)	32.5 (−41%)
Water use, m3	0.59	0.53 (−10%)	0.55 (−7%)	0.53 (−10%)	0.50 (−15%)	0.54 (−9%)	0.53 (−11%)
Land use, m2a	5.8	5.1 (−13%)	5.7 (−3%)	5.3 (−9%)	4.0 (−31%)	5.4 (−8%)	3.7 (−36%)
Acceptability							
∆diet (%)**	-	57	60	67	81	120	156

Detailed information on content of all 53 food groups in the optimized diets is available in Supplementary Table S3. Food amounts in raw weight. Optimization models: NNR2023, health constraints based on NNR2023 with no adjustments; Ruminant, additional lower limit for intake of ruminant meat (≥ observed intake); Legumes, lower limit for intake of pulses and legumes (≥40 g) and elements from the Danish FBDG (2020) (32) (fruit and vegetables ≥ 300 g each; total meat ≤350 g cooked weight /week); NNR2023+, NNR2023 scenario but with added incremental GWP constraints; Ruminant+, Ruminant scenario but with added incremental GWP constraints; Legumes+, Legumes scenario but with added incremental GWP constraints. ^†^Based on mean dietary intake data for adults 18–70 years from the Norkost 3 national dietary surveillance survey 2010–2011 (18). ^*^Percent change compared to the average observed diet. ^**^Total departure from observed diet (%).

### 3.2 Diets optimized to meet NNR2023 guidelines

Optimization of the observed diet to follow NNR2023 guidelines (NNR2023 scenario) resulted in an optimized diet with 9% lower GWP than the observed diet, and up to 14% reductions in other ICs (Table 3). Constraining ruminant meat intake to observed level in the Ruminant scenario led to an optimized diet with the same GWP as the observed diet, but with a 7% reduction in water use and 3% reductions in terrestrial acidification and land use. Imposing increased legume consumption (40 g/day) in the Legumes scenario led to similar reductions as seen in the NNR2023 scenario. Marine eutrophication increased by 6/7% for the Legumes and NNR2023 scenarios and 10% for the Ruminant scenario. Freshwater eutrophication also increased by 3% in the Ruminant scenario.

Content of main food groups (g/10 MJ) in the NNR2023, Ruminant, and Legumes scenarios is shown in Table 3. Detailed information on content of all 53 food groups is available in Supplementary Table S3. Content of bread is slightly lower than observed and nearly all white bread is switched out with wholegrain bread. The amount of other grains, cakes and cookies, and potatoes is nearly doubled compared to the observed diet. Content of vegetables, nuts, and plant-based fats is increased to meet NNR2023 guidelines, while the amount of fruit and berries is increased considerably above the guidelines. Dairy content (including cheese) is reduced by 37% from the observed diet, but at the upper limit of 500 g milk equivalents/day; further, dairy intake is re-distributed, with cheese decreasing by 88% and milk and other dairy increasing by 24 and 31%, respectively. Animal-based fats are decreased by 88%, but other discretionary foods (juice, beverages, sweets and snacks) remain in the diet in similar or lower amounts.

The three scenarios differ in regard to protein sources, though total content of main protein sources (g/day) is similar across scenarios. Content of fish, eggs, and white meat are the same in all three scenarios. In the Legumes scenario, legumes are at the lower limit of 40 g/day (+852%), while legumes are increased by only 2 g (+45%) in the other two scenarios. In the Ruminant scenario, content of ruminant meat is maintained at the observed level (62 g), while ruminant meat is decreased by about a third in both the NNR2023 and the Legumes scenario. Intake of pork is halved in both the NNR2023 and Legumes scenarios and is reduced by 84% in the Ruminant scenario. In all scenarios, processed meats are reduced by 80% and partially replaced by unprocessed meats.

### 3.3 Diets optimized to meet NNR2023 guidelines and GWP constraints

When imposing incremental GWP constraints to the NNR2023, Ruminant, and Legumes scenarios separately, the optimization algorithm was able to produce feasible outcomes up until a 30, 15, and 35% reduction in GWP, respectively, compared to the observed diet (Table 3). In all three scenarios, reductions in GWP coincided with reductions in other ICs, except for marine eutrophication, which increased 2% in the NNR2023 scenario and 8% in the Ruminant scenario, but decreased by 1% in the Legumes scenario. Overall reductions in IC categories were 2–4 times higher for the NNR2023 + and Legumes+ scenarios compared to the Ruminant+ scenario. Reductions were largest for terrestrial acidification, with a ~ 40% reduction for NNR2023 + and Legumes+, and a 13% reduction for Ruminant+. Land use also decreased by about a third in in NNR2023 + and Legumes+ but decreased by only 8% in Ruminant+. The decrease in water use was lesser and greatest in the NNR2023 + scenario (15%).

**Figure 1:**
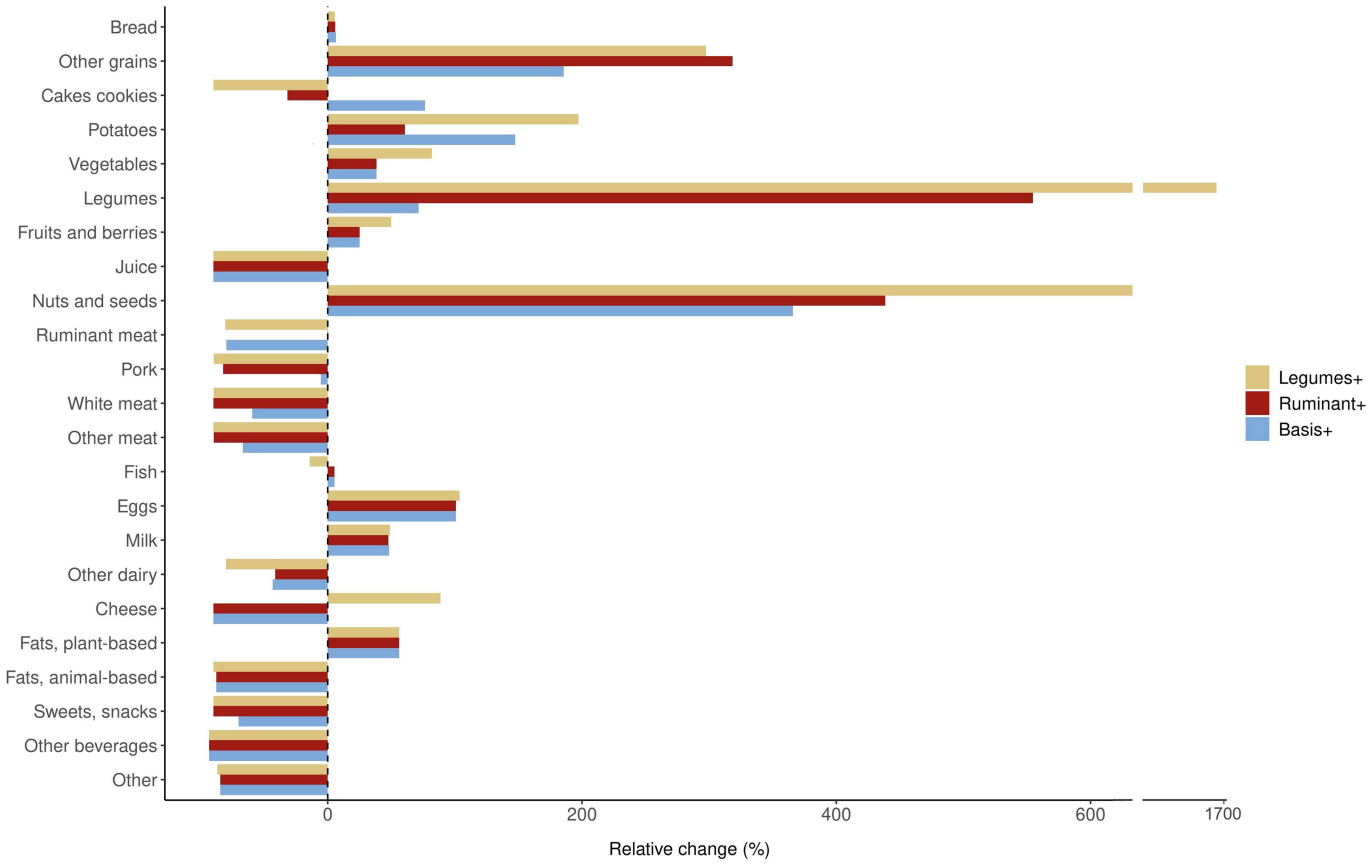
Relative changes (%) in content of food groups (g/10 MJ) compared to the average observed Norwegian diet^†^, after optimization of different dietary models to meet NNR2023 (6) health and nutrient guidelines, and incremental global warming potential constraints (0, −5%, −10, −15%…). Food amounts in raw weight. Optimization models: Legumes+ (−35% GWP), NNR2023 guidelines with additional lower limit for intake of pulses and legumes (≥40 g) and elements from the Danish FBDG (2020) (32) (fruit and vegetables ≥ 300 g each; total meat ≤350 g cooked weight/week); Ruminant+ (−15% GWP), NNR2023 guidelines with additional lower limit for intake of ruminant meat (≥ observed intake); NNR2023 + (−30% GWP), NNR2023 guidelines with no adjustments. ^†^Based on dietary intake data for adults 18–70 years from the Norkost 3 national dietary surveillance survey 2010–2011 (18).

Content of main food groups (g/10 MJ) in the NNR2023+/Ruminant+/Legumes+ scenarios is shown in Table 3 and Figure 1 provides a visual illustration of the relative changes (%) in food group amounts compared to the observed diet. Detailed information on content of all 53 food groups is available in Supplementary Table S3. Content of other grains is increased by 185% in NNR2023 + and by over 300% in Ruminant+ and Legumes+. NNR2023 + includes a daily portion of cakes and cookies, while Ruminant+ and Legumes+ include less of these foods. The amount of potatoes is 2–3 times higher than the observed diet, and highest in the Legumes+ diet. Content of fruit and vegetables is also highest in the Legumes+ scenario, increasing by 60% to meet the Danish FBDG. Content of dairy intake borders on the upper limit of 500 g milk-equivalents in all three scenarios, where cheese intake is decreased by 89% and redistributed to milk and other dairy sources. Animal-based fats are nearly removed from the diet and replaced with plant-based fats such as plant oils and margarine. Content of juice and both sugar-sweetened and artificially sweetened beverages are reduced by 90% in all diets, while alcohol is completely removed in line with NNR2023 recommendations. Sweets and snacks are reduced by 65% in the NNR2023 + diet and by 90% in Ruminant+ and Legumes+.

The three scenarios show key differences in sources of protein. Content of fish and eggs are the same in all three scenarios. White meat is reduced from the observed diet by 59% in NNR2023+, and by 89% in Ruminant+ and Legumes+. Legumes are increased from the observed diet by 71% in NNR2023+, 554% in Ruminant+, and 1705% in Legumes+. The relative changes are large due to low consumption of legumes in the observed diet; however, these changes represent increases of 3 g in NNR2023+, 24 g in Ruminant+, and 68 g in Legumes+. In Ruminant+, content of ruminant meat is maintained at observed level, while ruminant meat is decreased by 80% NNR2023 in NNR2023 + and Legumes+. Intake of pork is similar to the observed diet in NNR2023 + (56 g) but reduced by 84% in Ruminant+ and 90% in Legumes+. In all scenarios, the percentage of total meat that is processed is reduced by 80–90% compared to the observed diet.

### 3.4 Nutrient content of the optimized diets

Nutrient content of the optimized diets is provided in Supplementary Table S4. Limiting nutrients in the diet scenarios optimized to meet NNR2023 guidelines alone were similar and included saturated fat, sodium, vitamin A, folate, calcium, zinc, and selenium. Vitamin D was not constrained but was above the AR (7.5 μg) and below the RI (10 μg) in all three diets. Sodium, selenium (set to the AR, 65 μg) and zinc (set to the RI, 11.2 μg) were the strongest limiting constraints. All optimized diets contained the highest amount of sodium allowed by the model constraints (2,400 mg); this is partly due to the decision to maintain spices (including discretionary salt) at the observed level.

Limiting nutrients in the diets optimized to meet GWP constraints in addition to NNR2023 guidelines were in line with those listed above. Saturated fat and folate remained limiting nutrients in NNR2023 + but were not limiting in Ruminant+ and Legumes+. Zinc was limiting in NNR2023 + and Legumes+, but not in Ruminant+. Vitamin D was just above the AR in the NNR2023 + scenario, but below the AR in Ruminant+ and Legumes+. Sodium and selenium were the strongest limiting constraint in all scenarios.

### 3.5 Diet acceptability

The diet departure scores (Δdiet, as defined above) for the NNR2023, Ruminant, Legumes, NNR2023+, Ruminant+, and Legumes+ scenarios were 57, 60, 67, 81, 120, and 156%, respectively (Table 3).

### 3.6 Sensitivity analysis

In the first sensitivity analysis, constraints for all nutrients were lowered to the AR. Results are shown in Table 4. GWP reductions up to 40% were feasible for the NNR2023+/Legumes+ scenarios, but no further reductions were possible for the Ruminant+ scenario than in the main analysis.

**Table 4:** Sensitivity analysis.

	Observed diet^†^	NNR2023	Ruminant	Legumes	NNR2023+	Ruminant+	Legumes+
Bread	192	197	173	172	219	183	226
Other grains	40	70	77	70	162	130	164
Cakes, cookies	41	62	70	54	25	84	4
Potatoes	76	106	110	108	206	147	178
Vegetables	165	250	250	300	250	250	300
Fruit and berries	200	286	299	300	250	250	300
Milk	278	291	278	298	209	207	177
Other dairy	80	90	97	88	57	51	55
Cheese	43	16	17	16	9	10	12
Nuts and seeds	4	21	21	21	28	21	30
Legumes	4	5	5	40	14	7	40
Eggs	27	55	55	55	55	55	55
Fish	80	78	78	78	78	78	78
Ruminant meat	62	30	61	32	5	56	5
Pork	60	40	9	36	4	4	4
White meat	38	38	38	38	4	8	4
Other meat	5	2	2	2	1	2	1
Fats, plant-based	16	25	25	25	25	25	25
Fats, animal-based	16	6	6	8	2	2	2
Juice	120	100	100	100	12	12	12
Other beverages	397	317	325	320	26	119	26
Sweets, snacks	23	28	30	30	2	10	2
Water, coffee, tea	2,236	2,236	2,236	2,236	2,236	2,236	2,236
Other	32	4	4	4	4	4	4
Environmental measures							
Global warming potential, kg COe-eq	5.2	4.4 (−14%)*	5.1 **(−2%)**	4.5 **(−13%)**	3.1 **(−40%)**	4.4 **(−15%)**	3.1 **(−40%)**
Freshwater eutrophication, g P-eq	1.2	1.1 (−9%)	1.2 (+1%)	1.1 (−8%)	0.8 (−35%)	1.0 (−12%)	0.7 (−35%)
Marine eutrophication, g N-eq	4.9	5.2 (+6%)	5.4 (+11%)	5.1 (+5%)	5.0 (+2%)	5.4 (+10%)	5.0 (+2%)
Terrestrial acidification, g SO2-eq	55.0	43.3 (−21%)	52.7 (−4%)	43.8 (−20%)	28.9 (−47%)	46.4 (−16%)	28.9 (−47%)
Water use, m3	0.59	0.5 (−12%)	0.5 (−9%)	0.53 (−11%)	0.4 (−24%)	0.5 (−22%)	0.45 (−23%)
Land use, m2a	5.8	4.8 (−18%)	5.6 (−3%)	4.9 (−16%)	3.3 (−43%)	5.2 (−10%)	3.3 (−43%)
Acceptability							
∆diet (%)**	-	49	55	63	107	80	127

Content of main food groups (g/10 MJ) and environmental impacts (…) per 10 MJ in the average observed Norwegian diet and diet scenarios optimized to meet NNR2023 (6) health and nutrient guidelines and incremental global warming potential constraints (0, −5%, −10, −15%…) using average nutrient requirements (AR). Nutrient constraints for all nutrients were set to the AR for adults. Detailed information on content of all 53 food groups in the optimized diets is available in Supplementary Table S5. Food amounts in raw weight. Optimization models: NNR2023, health constraints based on NNR2023 with no adjustments; Ruminant, additional lower limit for intake of ruminant meat (≥ observed intake); Legumes, lower limit for intake of pulses and legumes (≥40 g) and elements from the Danish FBDG (2020) (32) (fruit and vegetables ≥ 300 g each; total meat ≤350 g cooked weight/week); NNR2023+, NNR2023 scenario but with added incremental GWP constraints; Ruminant+, Ruminant scenario but with added incremental GWP constraints; Legumes+, Legumes scenario but with added incremental GWP constraints. ^†^Based on mean dietary intake data for adults 18–70 years from the Norkost 3 national dietary surveillance survey 2010–2011 (18). *Percent change compared to the average observed diet. ^**^Total departure from observed diet (%).

Increasing the selenium constraint to the AI led to an 18% reduction in GWP for the NNR2023/Legumes scenarios, which is nearly double the GWP reduction seen in the main analysis (Supplementary Table S5). However, optimization results were feasible only up to a 20% reduction in GWP for the NNR2023+/Legumes+ scenarios, and a 5% reduction in GWP for the Ruminant+ scenario. Results were similar to the main analysis when the sodium constraint was updated to <2,300 mg, but the diets included less bread and more potatoes, breakfast cereals (NNR2023+) and legumes (Legumes+) (Supplementary Table S5). When the upper constraint on white meat was removed, white meat was doubled in both the NNR2023 and Ruminant scenarios, but unchanged in the Legumes scenario. GWP results were similar to the main analysis, with a 7% reduction in GWP in the NNR2023 scenario and a slight increase of 1% in GWP in the Ruminant scenario (Supplementary Table S5).

When ruminant meat was split into beef, lamb, and ruminant game (Supplementary Table S6), most of the ruminant meat in the Ruminant scenario was beef, while in the Ruminant+ scenario the majority was lamb. Much of the observed lamb intake was preserved in the scenarios without GWP constraints but was reduced by 90% in the NNR2023+/Legumes+ scenarios. Notably, land use was increased by 12% in the Ruminant+ scenario compared to the observed diet. Results did not differ dramatically when scenarios were run with and without a co-production constraint linking beef and dairy. Optimization scenarios also were run separately for four population sub-groups based on daily total meat intake (Q1:45 g, Q2:118 g, Q3:186 g, Q4:309 g) (Supplementary Table S7). In Q1, Q2, and Q3 it was possible to achieve a GWP reduction of 35% in the NNR2023+/Legumes+ scenarios, while only 30% was possible in Q4. Pork content was substantially lower in the optimized diets of Q1-Q3, but similar to the main analysis in Q4. The amount of lean fish was higher in all diets than in the main analysis.

## 4 Discussion

To our knowledge, this is the first study to assess quantitatively the environmental impact of optimized diets following the NNR2023 guidelines. We found that nutritionally optimized diets following NNR2023 guidelines had generally lower environmental impacts than the observed diet, but that outcomes were dependent on the distribution of meat consumption in the diet. Further, we were able to identify diets following NNR2023 guidelines up until a 3% reduction in dietary GWP; when a daily portion of legumes was added, a 35% reduction was feasible. Maintaining the observed level of ruminant meat (62 g/d) limited the GWP reduction to 15% and required greater dietary changes.

### 4.1 Optimized diets following NNR2023 guidelines

We found that optimizing the average observed Norwegian diet to follow NNR2023 guidelines alone (NNR2023 scenario) led to a diet with 9% lower GWP, decreased freshwater eutrophication, terrestrial acidification, water use, and land use, and increased marine eutrophication. Neither an increase in the intake of white meat or legumes had a notable impact on the environmental outcomes of following NNR2023 guidelines. Further environmental optimization of the diet resulted in nutritionally and environmentally optimized diets with up to with 30/35% lower GWP than the observed diet (without/with daily portion of legumes) and up to 41% reductions in the other measured environmental indicators, with the exception of marine eutrophication.

The optimized diets contained more grains and potatoes, nuts, fruit and vegetables, plant-based fats, fatty fish, and eggs, and less meat, cheese, animal-based fats, and discretionary foods than the average observed Norwegian diet. The main dietary changes involved the substitution of meat with cereals and potatoes, and the intra-category substitution of foods, particularly beef with pork in the meat category and cheese with milk and other dairy products in the dairy category. In the Legumes+ scenario, total content of meat is reduced by over 85% and replaced with a daily intake of pulses and legumes. Although decreased, a substantial amount of animal-based foods remained in the optimized diets (600–700 g/d) due to a number of factors including choice of objective function (i.e., minimizing distance from a diet already containing a large amount of animal-based foods), comprehensive nutrient constraints, and lower bounds for content of dairy products, fish, and ruminant meat. However, popular animal-based foods such as processed meat and fish products, cheese and other high-fat dairy products, and dairy fats were largely replaced with unprocessed meat and fish, low-fat milk and fermented dairy products, and plant-based margarines and oils. Consumption of discretionary foods such as sugar-sweetened beverages, alcohol, sweets, and snacks were also markedly decreased to comply with health guidelines and free up energy in favor of more nutrient-dense foods. These dietary changes align closely with those promoted by dietary guidelines; however, the acceptability of these measures on a consumer-level may be called into question.

These results suggest a synergy between health and environmental goals, in line with NNR2023’s mandate to integrate sustainability considerations into their health-based nutritional recommendations. However, although the majority of environmental measures improved after optimization to follow NNR2023 guidelines, marine eutrophication increased slightly, even after environmental optimization. This finding is in line with our previous research, where scenarios representing the Norwegian FBDG and the EAT-Lancet reference diet showed only minimal reductions in marine eutrophication compared to the observed Norwegian diet, while other impact categories were reduced dramatically (33). Increases in the amount of grains and vegetable oils contribute to this rise in marine eutrophication values. Previous evidence suggests that the high contribution of plant-based foods to levels of marine eutrophication necessitates substantial changes in dietary patterns in order to reach significant reductions and indicates a need for concurrent improvements in production methods (34, 35). Moreover, though water use is often highlighted in the literature as a potential trade-off in ‘sustainable’ diets (3, 36), our results indicate a decrease in water use when switching to healthier and/or more sustainable dietary patterns in Norway. These findings highlight the importance of considering national context when investigating sustainability.

The optimization model was able to identify feasible diets up until a 35% reduction in GWP, and a 40% reduction in GWP if nutrient constraints were lowered to the AR for adults. Previous studies generally measure climate impact in terms of greenhouse gas emissions (GHGEs). A number of these studies have discovered feasible optimized diets at large GHGE reductions (>50%), with some identifying diets at over 70% reductions in GHGE (15, 37–46). However, many of these studies focused on nutrient recommendations only, and included few or no epidemiology-based targets for food groups (39, 41–46). In addition, many of the optimized diets included increased amounts of legumes and fortified plant-based meat and dairy substitutes (38, 39, 41, 43, 44). These foods provide nutrients such as protein, vitamins B12 and D, calcium, iron, and selenium, at a lower environmental impact, and thus often replace animal-based foods in optimized diets (23). Consumption of these foods is minimal in Norway and thus less likely to be modified markedly by the quadratic optimization model. Excluding these foods due to low observed consumption, while making widespread reductions in content of meat, animal-based fats, and discretionary foods, likely oversimplifies consumer acceptability. Use of nutrient supplements (e.g., vitamin and mineral pills, fish oils) could also ensure that nutritional needs are met as animal-sourced foods are removed from the diet, allowing for greater GHGE reductions. Nonetheless, there is a general consensus that GHGE reductions beyond 30–40% may result in impaired nutritional adequacy or require large dietary shifts that may compromise acceptability (3, 46–48). A GHGE reduction of ~30% corresponds also to the GHGE level of the Danish plant-rich diet, which lays the foundation for the existing FBDG in Denmark, and to that of the optimized diet designed for Denmark by Nordman et al. (24).

The optimized diet for Denmark (24) had a similar but slightly lower content of animal-sourced products (~550 g) than in the present study, but these foods were differently distributed. The diet included more total meat, poultry, cheese, and bread, but less beef, egg, milk, fish, grains, and potatoes than the NNR2023 + scenario diet proposed in the present study. These differences partially reflect cultural differences resulting from national agricultural strategies; however, the differences are likely also due to methodological variations such as source of nutritional and environmental data, and choice of constraints. Nonetheless, the dietary changes observed in the present study resemble results from previous studies performed in other Nordic and European settings (3, 15, 24, 41, 42, 47). Other optimization studies have also seen a redistribution between ruminant meat and pork and/or poultry (15, 42, 44, 49). Both Grasso et al. (49) and Kesse-Guyot et al. (15) found that total meat content of the optimized diets remained stable up even at a 50% reduction in GHGE, but that meat was strongly redistributed at the cost of ruminant meat. This redistribution is driven by, e.g., the compromise between satisfying nutritional constraints for zinc, iron, and sodium, and the environmental constraints (15). In the present study, nutritional constraints for selenium, zinc, and sodium were drivers of the redistribution of meat sources and the maintenance of a considerable red meat intake in the NNR2023 + scenario. Forcing the model to include legumes in the diet in the Legumes+ scenario did reduce the amount of meat included in the diet considerably and improve environmental outcomes. Furthermore, previous studies in Nordic countries have reported large increases in the amount of cereals and potatoes in optimized diets (24, 41, 42).

### 4.2 The case of ruminant meat

Another central aim of this analysis was to assess the possibility of maintaining ruminant meat consumption at the observed level in the Norwegian diet, while simultaneously reducing dietary environmental impact. We found that maintaining daily ruminant meat intake at 62 g (equivalent to ~300 g cooked meat per week) eliminated the reduction in GWP seen when adjusting the diet to follow NNR2023 guidelines and increased both freshwater eutrophication and marine eutrophication. However, the adjustment still led to small reductions in terrestrial acidification, water use, and land use. If intake of white meat was simultaneously allowed to surpass the observed level, environmental impact increased for all indicators except water use. When imposing incremental GWP reductions, we found that the model could not produce feasible diets at the observed level of ruminant meat consumption beyond a 15% reduction in GWP, even if nutrient constraints were lowered to the AR for adults.

The dietary changes required to induce a 15% GWP reduction for the Ruminant+ scenario diet were similar to those seen for the NNR2023 + scenario at a 30% GWP reduction. That is to say, due to the high contribution of ruminant meat in the Ruminant+ scenario to its overall environmental impact, extensive dietary changes were necessary to elicit even small reductions in GWP. These changes include large increases in the amount of grains, legumes, and seeds, decreases in white meat and cheese, and differential distribution within the fruit and vegetables categories. Again, processed meats were replaced with unprocessed meats, and discretionary foods were virtually removed from the diet. Moreover, in the Ruminant+ scenario, both total meat and pork were substantially reduced, by 53 and 82%, respectively, to satisfy the upper limit for red meat consumption. In other words, reaching a 15% GWP reduction while maintaining 62 g of ruminant meat in the diet requires the near elimination of other meat types.

Since pork is an important source of zinc and selenium in the optimized diets, legumes were increased by 600% to 28 g/day to meet nutrient constraints in the Ruminant+ scenario. This is an example of an acceptability trade-off: low legume consumption in the observed diet indicates that large increases in legume content are less acceptable to consumers than increases in other more frequently consumed foods and potentially unrealistic. However, for population groups intent on maintaining their consumption of ruminant meat, increasing legumes and heavily decreasing intake of other meat types may be a more acceptable dietary change than decreasing ruminant meat. One study from China sought to identify food groups with less consumption elasticity, arguing that respect for food culture in optimization modeling should go beyond minimizing changes in amount of all foods (50). However, in line with our results, Yin et al. found that inclusion of additional cultural criteria impeded the environmental benefits.

### 4.3 Sustainability goals

Compared with environmental boundaries downscaled from the EAT-Lancet targets for 2050 using an equal per capita approach as described by Wood et al. (51), even the scenario with the greatest GWP reduction (40%) has a carbon footprint double the target for greenhouse gas emissions (tCO_2_-eq/yr). The degree of change needed to reduce environmental footprint of Norwegian consumption below the environmental boundaries is particularly large for carbon footprint and will entail even more drastic dietary changes than those of the diets outlined in this paper. Still, the diets align with the advice of the Norwegian Environment Agency. In their calculations, the Norwegian Environment Agency report that if the entire Norwegian population follows the dietary advice for red meat and replaces the reduced amount of meat with plant-based food and fish, there is a reduction potential of 2.9 million tonnes of CO_2_-equivalents in the period 2021–2030 (5, 52). These dietary changes in addition to reduction of food waste and an increase in the proportion of Norwegian-produced food consumed represent the main governmental strategies to decrease emissions from the agricultural sector (5).

### 4.4 Strengths and limitations

Quadratic optimization is a data-driven method that is highly sensitive to methodological choices, as well as to the input data and its uncertainties. Our comprehensive approach to the healthiness of the diet by inclusion of epidemiology-based targets for food groups, in addition to nutrient criteria, is a strength of the study. However, the results are sensitive to our interpretation of the written NNR2023 guidelines. For example, we have chosen a conversion factor of 1:10 for cheese to milk in this study, while the NNR2023 committee provide a range equivalent to 1–2 grams of cheese per 10 g of milk. Other examples include the exclusion of juice from the fruit recommendation, upper limit for egg intake, and vegetable oil recommendation. Nevertheless, we believe that our interpretation is representative of the overall pattern of consumption recommended by NNR2023. As suggested by Gazan et al. (53), we used 53 food sub-groups rather than the original 1,507 food items as decision variables in the optimization models. This method is often preferred in studies intended for public health purposes as it guarantees a variety in the underlying food items and allows for easier communication of results (24, 53). Limiting number of food sub-groups while simultaneously imposing comprehensive nutrient and health-based constraints limited the solution space and led to an array of optimized diets that share the same overall structure.

Further, quadratic models penalize large deviations and thereby tend to generate relatively small changes to many separate foods; foods that are consumed in very small amounts in the observed diet are less likely to be modified markedly by the optimization model. Willingness to make changes in consumption of different foods is often dependent on cultural factors beyond current intake; it is therefore a strength of the present study that we have highlighted the cultural importance of ruminant meat in the Norwegian diet, building on the study by Yin et al. (54) and increasing the relevance of our results for the Norwegian setting. Still, future research should explore the use of weighting factors based on indicators of people’s readiness to make changes in intake of different food groups. Using individual diets, rather than population averages as the optimization variables is another method of accounting for individual variability in dietary patterns, needs, and preferences. With this method, constraints such as nutrient requirements can also be set at the individual level, ensuring that the needs of certain groups (i.e., iron requirements for menstruating women) are met.

The quality and uncertainties of the dietary intake, nutrient, and environmental impact data are important limitations that influence the reliability of the results. While the dietary data used in the present study are of a high quality and national representativeness, all dietary data is subject to limitations, such as misreporting and selection bias (55). The results from the newest Norwegian dietary surveillance survey (2022–2023) were not available at the time of this work, so data from the previous survey (2010–2011) were used (18, 56). Comparisons of results from the two surveys show a decrease in the intake of wholegrain bread, fruit, potatoes, lean fish, and low-fat milk, while the intake of vegetables, cheese, sugar/sweets and sugar-free soft drinks has increased (56). Results also show a slight increase in meat consumption, specifically ground meat and white meat, while the intake of red meat has decreased slightly. These results indicate undesirable trends in Norwegian dietary behavior, and further challenge the acceptability of the optimized diets proposed in this paper.

It is a strength of this study that six environmental indicators were included, and inclusion of a co-production factor, often overlooked in similar studies, increased representativeness of the Norwegian food system. Still, environmental data based on life cycle analysis values involves uncertainties (i.e., differences in the methods applied, year of data collection for primary production, standard factors used, exclusion of avoidable food losses, etc.). Water use values were not adjusted for water scarcity due to a lack of data and may underestimate water consumption of certain foods. Further, although the environmental database used in the present study contains values for all 53 food sub-groups, we assigned environmental values to 38 aggregated environmental groups to reduce uncertainty stemming from food sub-groups with more uncertain environmental values. The grouping of foods involved a series of decisions that were driven by knowledge of the Norwegian context, but ultimately subjective. Moreover, the co-production factor was linked to the food sub-group ruminant meat, as opposed to beef specifically. This affected environmental values for the sub-group and eliminated in practice content of lamb/mutton in the optimized diets. Finally, we recognize that there exist several other dimensions of sustainability that were beyond the scope this study, including biodiversity and social, economic, and animal welfare concerns such as cultural landscape values and self-sufficiency. Data-driven methods also fail to capture social and psychological drivers of food habits, e.g., cultural practices, meal sharing, taste, convenience, and food literacy (57).

## 5 Conclusion

Our findings indicate that the NNR2023 guidelines outline diets that have generally lower environmental impacts than the observed average Norwegian diet. Diets that are nutritionally adequate with considerably reduced GWP can be achieved for Norwegian adults but will require substantial dietary changes that may challenge consumer-level acceptability, including large reductions in intake of meat and discretionary foods and beverages. Daily consumption of legumes could further increase environmental benefits but entails the development of new dietary habits. Moreover, we found that the possibility of reducing environmental impact of the diet while retaining the observed intake of ruminant meat (62 g/d) is limited and presupposes a willingness to make substantial changes in intake of other foods.

In summary, increased compliance with NNR2023 guidelines has the potential to increase both population health and environmental sustainability and should be promoted to all Norwegian adults. Our findings contribute to the ongoing work of defining sustainable dietary patterns for Nordic countries. Future research should focus on expanding optimization models to include other aspects of sustainability to better capture the complexity of food systems. This research may also consider the use of individual optimization models that account for the needs and preferences of different population groups.

## Data Availability

The data analyzed in this study is subject to the following licenses/restrictions: this study used third party dietary data made available under licence that the author does not have permission to share. The integrated environmental and nutritional database is maintained by the Department of Nutrition at the University of Oslo. Requests to access these datasets should be directed to JL, j.m.lengle@medisin.uio.no.
